# The Accuracy of Cytology, Colposcopy and Pathology in Evaluating Precancerous Cervical Lesions

**DOI:** 10.3390/diagnostics12081947

**Published:** 2022-08-12

**Authors:** Liana Pleş, Julia-Carolina Radosa, Romina-Marina Sima, Radu Chicea, Octavian-Gabriel Olaru, Mircea-Octavian Poenaru

**Affiliations:** 1 Department of Obstetrics and Gynecology, Carol Davila University of Medicine and Pharmacy, 050474 Bucharest, Romania; 2 Bucur Maternity, Saint John Hospital, 012361 Bucharest, Romania; 3 Department for Gynaecology, Obstetrics and Reproductive Medicine, Saarland University Hospital, Kirrberger Straße 100, Building 9, 66421 Homburg, Germany; 4 Faculty of Medicine, Lucian Blaga University of Sibiu, 550024 Sibiu, Romania

**Keywords:** colposcopy, cervical cancer, screening, biopsy, accuracy, audit

## Abstract

**Introduction:** Cervical cancer (CC) is the third most common cancer in the world, and Romania has the highest incidence of cervical cancer in Europe. The aim of this study was to evaluate the correlation between cytology, colposcopy, and pathology for the early detection of premalignant cervical lesions in a group of Romanian patients. **Methods:** This observational type 2 cohort study included 128 women from our unit, “Bucur” Maternity, who were referred for cervical cancer screening. Age, clinical diagnosis, cytology results, colposcopy impression, and biopsy results were considered. Colposcopy was performed by two experienced examiners. The pathological examination was performed by an experienced pathologist. **Results:** The cytology found high-grade squamous intraepithelial lesions in 60.9% of patients, low-grade squamous intraepithelial lesions in 28.1%, atypical squamous cells for which a high-grade lesion could not be excluded in 9.4%, and atypical squamous cells of undetermined significance, known as *repeated* LSIL, in 1.6%. The first evaluator identified low-grade lesions in 56.3%, high-grade lesions in 40.6%, and invasion in 3.1% of patients. The second evaluator identified low-grade lesions in 59.4%, high-grade lesions in 32.0%, and invasion in 8.6% of patients. The pathological exam identified low-grade lesions in 64.1%, high-grade lesions in 25%, and carcinoma in 14% of patients. The colposcopic accuracy was greater than the cytologic accuracy. **Conclusions:** Colposcopy remains an essential tool for the identification of cervical premalignant cancer cells. Standardization of the protocol provided an insignificant interobserver variability and can serve as support for further postgraduate teaching.

## 1. Introduction

Cervical cancer (CC) is the third most common cancer in the world, and Romania has the highest incidence of cervical cancer in Europe [1,2]. Programs designed to reduce cervical cancer morbidity and mortality are based on the early detection of premalignant lesions. Improved cervical cytology and human papillomavirus (HPV) screening methods have decreased cervical cancer incidence [3,4]. A recent report indicated the high reliability of novel droplet digital PCR in molecularly characterizing premalignant uterine cervical lesions by detecting viral DNA belonging to different HPV genotypes [5]. However, in Romania, many patients are still diagnosed with late-stage cervical cancer despite the consistent introduction of national health programs for early detection that have been implemented in recent years [6,7].

In Romania, procedures such as HPV testing, colposcopy, and biopsy are used to diagnose abnormally classified cervical lesions, as proposed by the American Society for Colposcopy and Cervical Pathology (ASCCP) [8]. Although most practitioners follow these guidelines, there are no standardized guidelines or audit processes for performing colposcopies in our country. Only gynecologists who are trained and certified are legally allowed to perform colposcopies, but there is a wide variability in interpretation and management, leading to under- or overdiagnosis and treatment.

The present study aimed to evaluate (representing an internal audit) the performance of colposcopic examination in our unit by examining the correlation between cytology (Bethesda 2001 terminology [6]), colposcopic appraisal, and biopsy histology for the early detection of premalignant and malignant cervical lesions.

## 2. Material and Methods

This was an observational cohort study carried out between January 2018 and April 2020 in the “Bucur” Obstetrics and Gynecology Clinical Department at “Saint John” Hospital. Our unit is a university tertiary center that provides primary evaluation of and second opinions on cervical pathology and acts as a mandatory teaching center for physicians to become certified in colposcopy. The study was intended to be conducted over three years, but the unit was closed to the general population during the COVID-19 pandemic period in April 2020.

### 2.1. Study Participants

The study participants were women referred to our center for cervical screening following the implementation of the National Cervical Cancer Prevention Screening Program. The patients underwent cervical smear tests according to the program specifications after informed consent was obtained and after relevant clinical data had been registered and identified. The study was approved by our ethical board (1347/05.12.17). Patients with the following abnormal cytology types were included in the study: ASC-US (*atypical squamous cells of undetermined significance*), ASC-H (*atypical squamous cells for which a high-grade lesion cannot be excluded*), LSIL (*low-grade squamous intraepithelial lesion*), and HSIL (*high-grade squamous intraepithelial lesion* or inflammatory result). All patients received a colposcopy examination and biopsy and histology if necessary. We only included the above-mentioned lesions to simplify statistical correlation.

We designed a study based on abnormal cervical cytologic results. Women between the ages of 21 and 65 with cytologic results of ASC-H, LSIL, HSIL, or ASC-US followed by LSIL were included. Those who had undergone previous cervical surgical interventions were excluded.

### 2.2. Clinical Examination

Cytologic results were classified as benign lesions, LSIL, HSIL, and invasive cancer. A punch biopsy was taken from the most suggestive parts of the lesion.

Colposcopies were randomly performed for all of the included patients, and results were validated by two certified and experienced evaluators (L.P. and M.O.P.) with 15 and 10 years, respectively, of colposcopy examination and teaching experience. The pathological examination was performed by a single experienced pathologist.

Colposcopic examination protocol was standardized at 15× magnification of as follows: cervical examination without preparation, application of 5% acetic acid for 1–2 min, Lugol’s iodine test.

For colposcopic evaluation, we used the terminology according to international nomenclature [7]. Iodine uptake was noted as brown (positive uptake), yellow-brown (partial uptake), and mustard yellow (no uptake). The colposcopic data results were coded as low-grade lesions, high-grade lesions, or invasive lesions for statistic variable interpretation and analyses.

We registered the age, clinical diagnosis, cytology results, colposcopic impression, and biopsy results of all of the patients in an Excel spreadsheet. The most relevant figures for each step of the colposcopic examination were recorded and stored for cross examination by the second evaluator, who was blinded to the appraisal of the first evaluator.

Cervical biopsy specimens were interpreted using standard histologic descriptions as nondysplastic biopsies (normal, cervicitis, para/hyperkeratosis without cervical intraepithelial neoplasia, microglandular hyperplasia, metaplasia with or without atypia, reactive changes with or without atypia, or benign inflammation changes), low-grade biopsies (cervical intraepithelial neoplasia 1, HPV changes/effect, or HPV excluded), high-grade biopsies (cervical intraepithelial neoplasia 2 or 3 or carcinoma in situ), and invasive cancer. All pathological lesions were classified as low-grade lesions, high-grade lesions, or carcinoma. After biopsy, patients were managed according to the ASCCP guidelines [8].

To reduce the bias and uniformity and the objectivity of the colposcopic examination results, we did not mention HPV status. This test is not possible in our hospital, and the patients come with a result from another clinic, and very often, these documents are not available. Another aspect that we excluded was the HPV vaccination status of the patients. We do not have a national program, so the number of women who have received a vaccine is limited, especially because the costs are significant.

The main outcome of the study was the correlation of the cytology, colposcopy, and pathology results. The secondary outcome of the study was interobserver variability. In order to reduce bias, the evaluators agreed on the standard examination protocol, magnification size, and figure storage.

### 2.3. Statistical Analysis

Statistical analyses were performed with SPSS version 23.0 software (SPSS Inc., Chicago, IL, USA). Data are expressed as means ± standard deviation or numbers of patients. The χ^2^ test or Fisher’s exact test was performed to compare patient characteristics. An unpaired *t*-test was used to compare variables with a normal distribution. A *p*-value < 0.05 was considered statistically significant. This was a type 2 cohort study, and the missing values were excluded from the beginning of the statistical analyses.

## 3. Results

**Figure 1:**
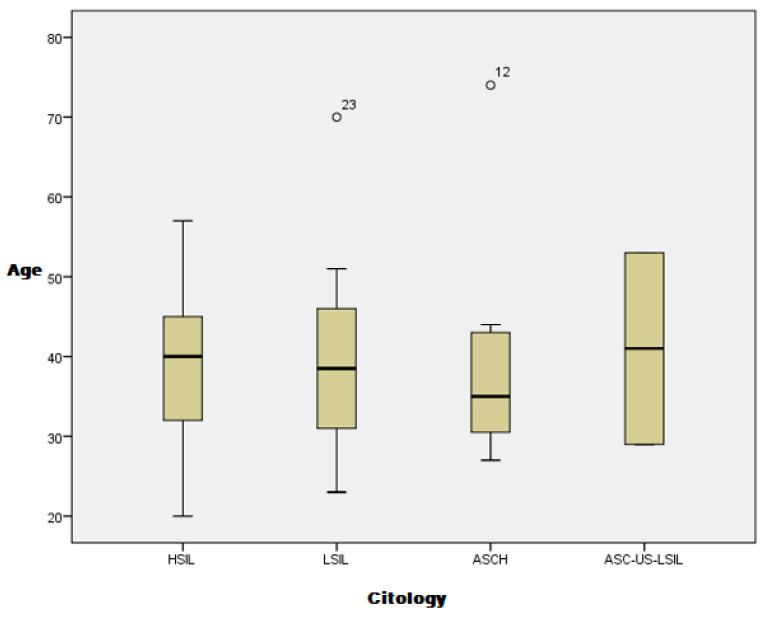
Cervical cytology distribution by patient age.

The study included 150 patients with abnormal cervical cytologic results. Six patients declined to take part in the study, and twelve patients did not return for colposcopic evaluation after cervical cytology smear samples were taken. Four patients with unsatisfactory colposcopy results were excluded from the study. Finally, the study cohort comprised 128 patients with cytologic, colposcopic, and cervical biopsy results. All of the patients were white (Caucasian), and the mean age was 38.95 years (confidence interval [CI]: 37.22–40.57). The mean ages of the patients with HSIL, LSIL, and ASC-H were 38.96 years (CI: 36.94–40.98), 38.82 years (CI: 35.45–42.20), and 38.33 years (CI: 30.31–46.35), respectively. The cytologic results were not influenced by the patient’s age in our study (Figure 1).

HSIL was the most frequent cytological result (60.9%) followed by LSIL (28.1%), ASC-H (9.4%), and ASC-US–repeated LSIL (1.6%). The colposcopic evaluations performed by the first evaluator revealed low-grade lesions in 56.3% of cases, high-grade lesions in 40.6% of cases, and invasion in 3.1% of cases. The colposcopic results obtained by the second colposcopic evaluator were low-grade lesions in 59.4% of cases, high-grade lesions in 32.0% of cases, and invasion in 8.6% of cases. We identified correspondence between the cytologic results and the colposcopic examinations for evaluator 1 and evaluator 2 (Table 1).

For the first evaluator, 57.69% of the cytological HSIL results were assessed as being high-grade lesions, 0.05% were assessed as invasive lesions, and 37.17% were assessed as low-grade lesions. The colposcopic accuracy was greater in the low-grade lesions for the first evaluator; 94.44% of the cytological LSIL results were evaluated as low-grade lesions by the colposcopy evaluator. Of the cases with HSIL cytology, the second evaluator identified high-grade lesions in 43.58%, invasion in 14.10%, and low-grade lesions in 42.30%. In the LSIL cytologic group, the second evaluator was also more aligned with the colposcopic results; low-grade lesions were identified in 88.89% of cases, and high-grade lesions were identified in 11.11%. Therefore, there were differences between the two experienced colposcopic evaluators for all of the lesion types, but this was the most evident for high-grade lesions (Table 2).

**Figure 2:**
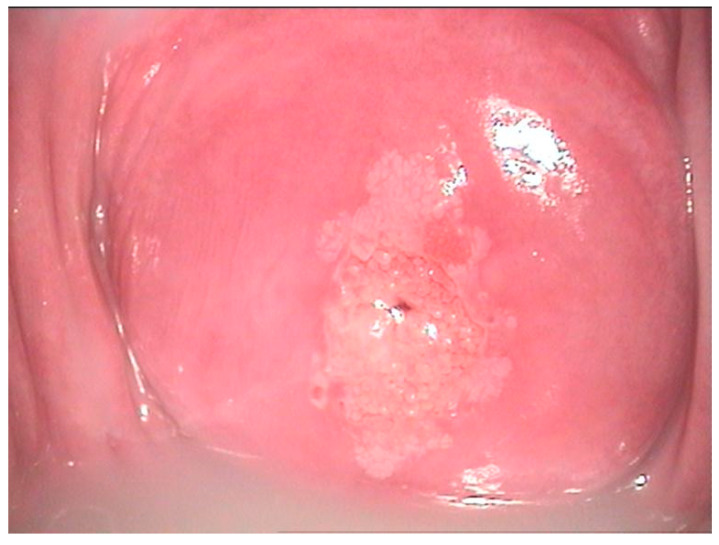
Colposcopic mage of a low-grade LSIL PAP smear. At the border of the ectopic epithelium, both anterior and posterior fine acetowhite epithelium with fine mosaic is noticed. At 7 o’clock, a colleret gland is present.

**Figure 3:**
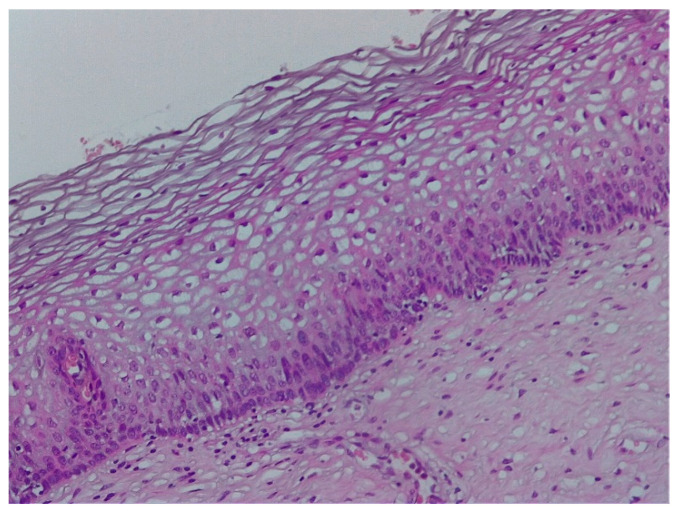
Histologic correspondent of the previous case—LSIL basal and parabasal cell proliferation, superficial epithelial thickening and acanthosis with frequent koilocytes in the intermediary layer (hematoxylin–eosin 10×).

**Figure 4:**
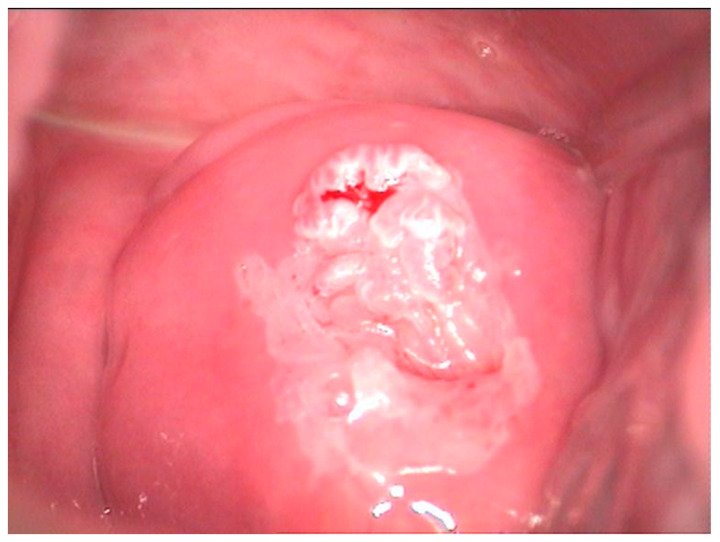
Colposcopic image after acetic acid application indicates aceto-white overriding epithelium on the entire anterior lip, suggesting condiloma acuminata. The lesion is ascending in the cervix, and its distal limit cannot be assessed.

**Figure 5:**
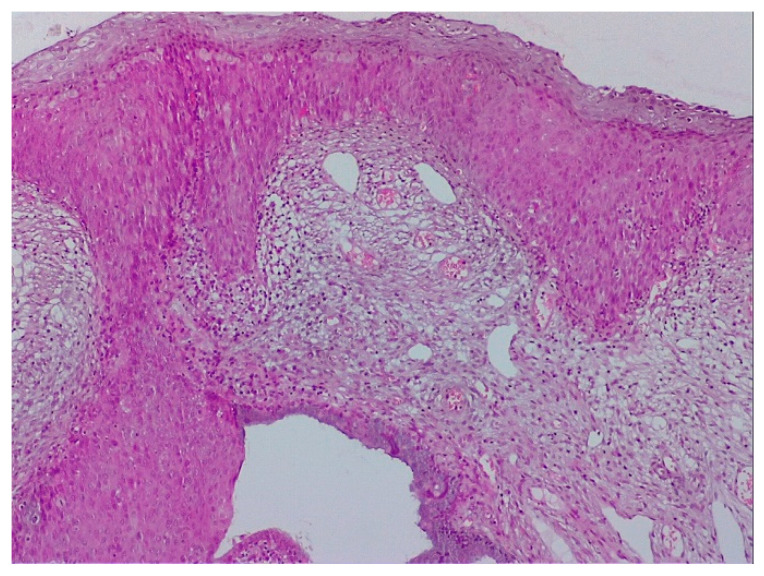
CIN 2—histological specimen after biopsy of the previous case. We notice a condiloma-like aspect of the Malpighian epithelium, the loss of nuclear polarity in two-thirds of the epithelial thickness, and a lot of koilocytes in the intermediary layer and ulcerate junction.

**Figure 6:**
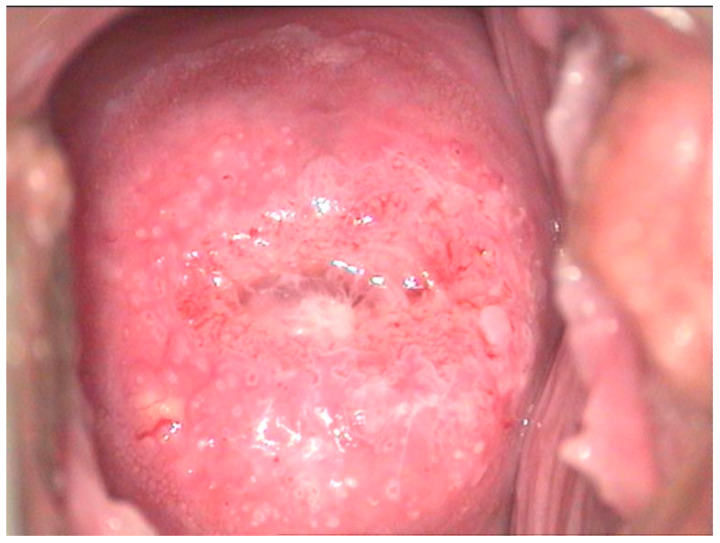
Polymorphic colposcopic image—circumferential aceto-white epithelium with intraglandular squamous transformation, papillary proliferation on the anterior lip, and peripheric fine mosaic; ASCUS PAP smear.

**Figure 7:**
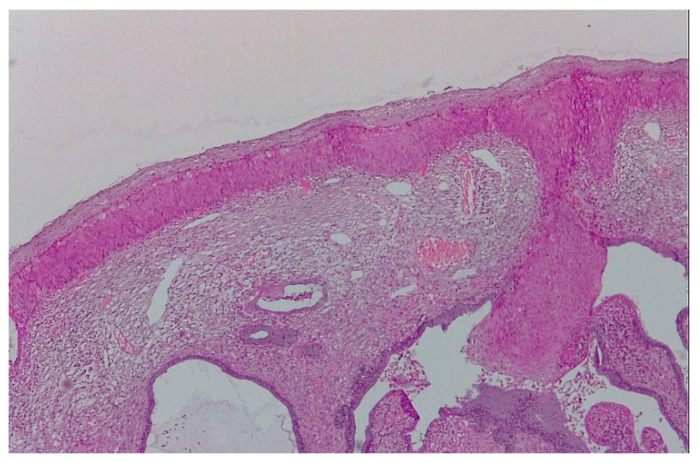
Histological correspondence of the case above, CIN2 lesion: image containing squamous papillary epithelium with many koilocytes and intraglandular mature metaplasia (hematoxylin–eosin 5×).

**Figure 8:**
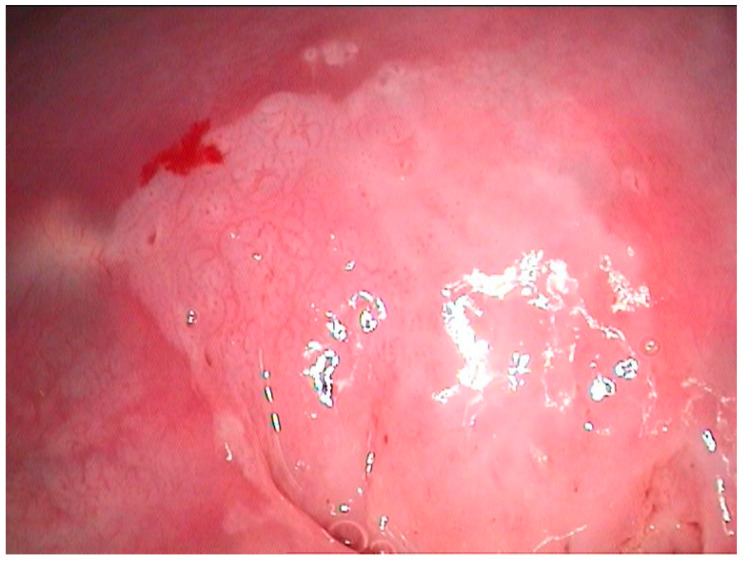
HSIL—colposcopic view of a HSIL PAP showing extensive aceto-white epithelium without a visible border at the distal end (intracervical) with mosaic and punctuation at 11 o’clock.

**Figure 9:**
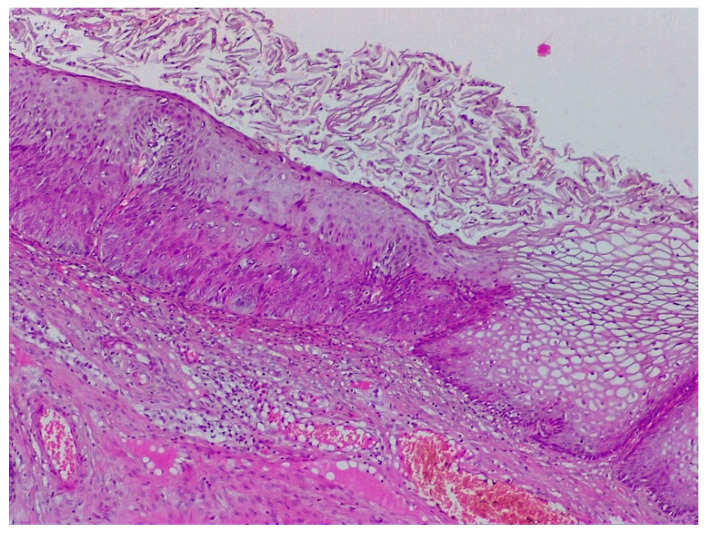
Histological specimen of the previous case illustrating CIN 3 lesion—proliferation of squamous cells with abnormal mitosis throughout the entire thickness and acanthosis and parakeratosis of the superficial layers. Right: Koilocyte transformation illustrating the simultaneous presence of two lesions of different severity levels. (Hematoxylin–eosin 10×).

**Figure 10:**
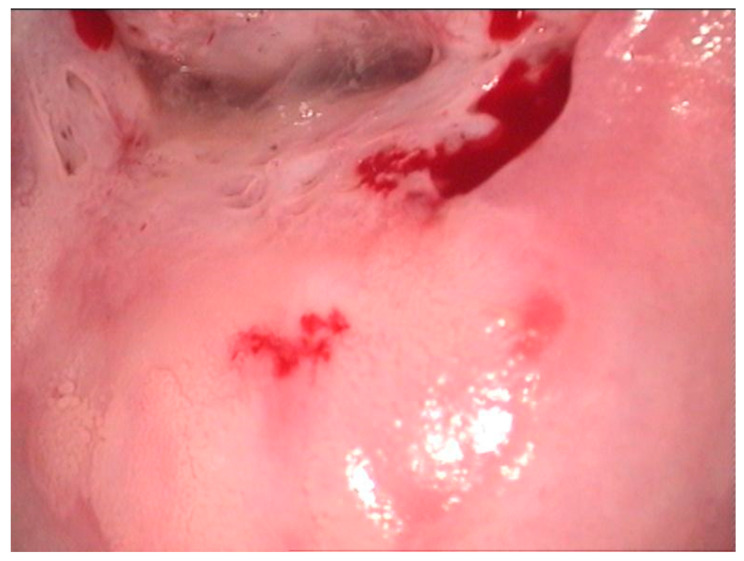
Colposcopic image of high-grade lesion revealed by coarse aceto-white epithelium on the posterior lip depicting mosaic, petechial junctional zone, and glandular criptae.

**Figure 11:**
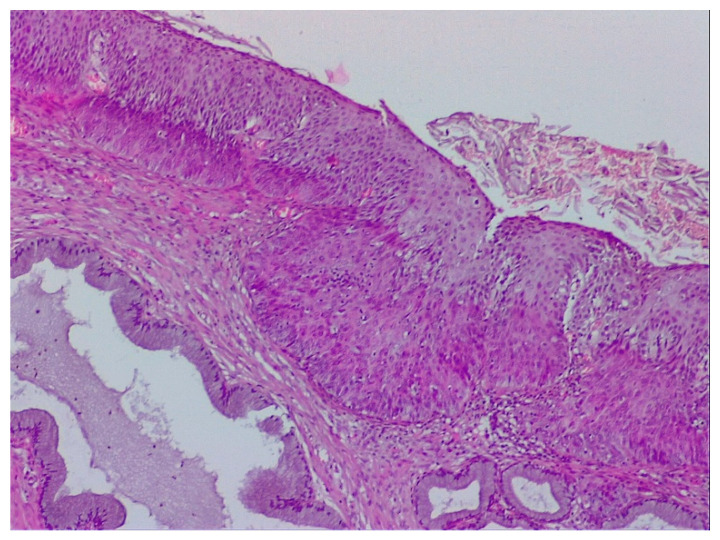
Image showing three typical aspects of CIN in the case in Figure 8: squamous abnormalities and glandular dilatations as well as moderate ulceration of the squamo–cylindric junction with anisocoria and nuclear polymorphism. (Hematoxylin–eosin 10×).

**Figure 12:**
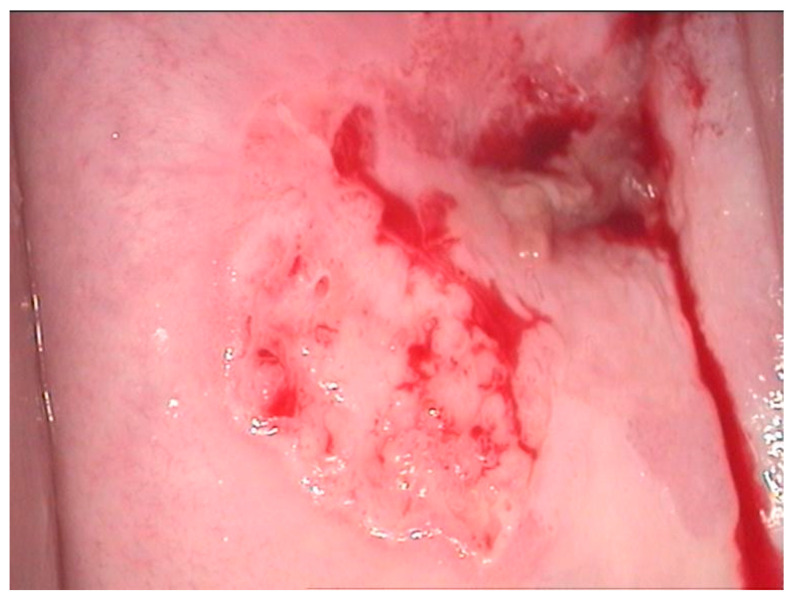
Colposcopic image of a HSIL cytology result depicting aceto-white epithelium at 7 o’clock with abnormal irregular and petechial vascularization that is highly suggestive of an invasive lesion.

**Figure 13:**
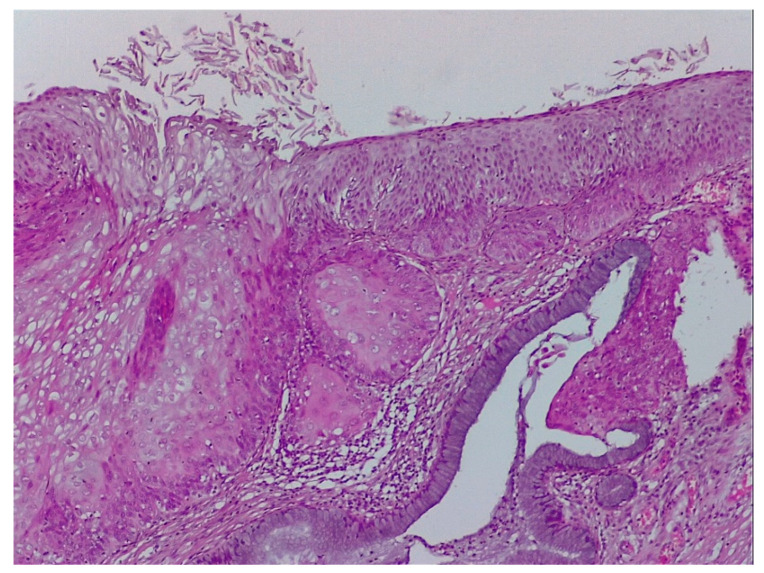
Pathological specimen of the case described in Figure 9: microinvasive carcinoma, superficial ulceration, broken basal membrane, and presence of the keratinization foci in stroma. The columnar epithelium is normal. (Hematoxylin–eosin 10×).

The pathological exam identified low-grade lesions in 64.1% of cases (Figure 2, Figure 3, Figure 4, Figure 5, Figure 6 and Figure 7), high-grade lesions in 25% of cases (Figure 8, Figure 9, Figure 10 and Figure 11), and carcinoma in 10.9% of cases (Figure 12 and Figure 13). For low-grade lesions, the accuracy of the two observers was 80.48% and 82.92%, respectively. The difference is the result of four cases that the first evaluator identified as high-grade lesions and that the second evaluator identified as high-grade lesions (two cases) and invasion (two cases). The colposcopic examination accuracy was similar for both evaluators regarding high-grade lesions. The second evaluator underestimated two cases differently than the first evaluator. For carcinoma, both evaluators identified one case as a low-grade lesion.

The correspondence between the cytology and pathology results can be found in Table 3. No LSIL cytology was diagnosed as carcinoma, and 13 cases were pathologically classified as high-grade lesions. One case of ASC-H was pathologically classified as carcinoma.

## 4. Discussion

Cervical cancer (CC) is the fourth most frequent cancer in women worldwide. HPV infection is associated with the majority of CC cases, but a small proportion of CCs actually test negative for HPV [9]. In the age of HPV-based screening for cervical cancer, the place of colposcopy and its sensibility and specificity in detecting severe premalignant or invasive cervical lesions can be challenged. We aimed to demonstrate that there is a good correlation between the three tools for cervical lesion detection, as cytology can detect, colposcopy can identify, and pathology can diagnose the disease.

The accuracy of colposcopic examination was good for all types of cytologic results, but it was the highest for low-grade lesions. The pathologic results demonstrated the accuracy of colposcopic examination among the two evaluators regarding low-grade and high-grade lesions. The accuracy for low-grade lesions was 80.48% and 82.92% for evaluators 1 and 2, respectively. The colposcopic examination accuracy was similar for both evaluators regarding high-grade lesions. We conclude that in our study, the colposcopic accuracy was greater than the cytologic accuracy.

Our results are similar to those in the literature regarding the accuracy of colposcopy impression and the diagnosis of high-grade squamous lesions. Iodine-negative areas include the columnar epithelium (lacking glycogen), peripheral areas with patchy uptake due to cervicitis, and immature metaplasia [10]. Mustard yellow areas with distinct borders suggest more severe disease [11]. Colposcopy assessment more often overestimates the severity of lesions; however, given the minor side effects of biopsy and the substantial morbidity that may follow a false-negative, and detection of HPV may prevent unnecessary surgical procedures, especially with ASCUS [12]. Because the colposcopic diagnosis of pre-invasive cervical carcinoma has a sensitivity ranging from 79% to 82% and specificity ranging from 73% to 87% [13], keeping colposcopy in the gynecologist’s toolbox is advisable, and new colposcopy approaches are being developed [14].

Many studies have aimed to compare the accuracy and differences between colposcopic evaluators. One study tried to examine the interobserver differences regarding in vivo and recorded images of the transformation zone of the cervix. In vivo examination has greater sensitivity due to local changeable factors, such as the patient’s position or the magnification of the cervix [15]. Another similar study concluded that computer imaging or even smartphones are advantageous for colposcopy being carried out by inexperienced colposcopists because of storage and quality control capabilities [16]. Although we had two colposcopic evaluators (one in vivo and one for computer-saved images), there was no significant difference between the two. This may be because both are experienced evaluators.

Currently, tests and procedures such as colposcopy and biopsy are applied to manage various cytologies [17]. While these techniques may cause patient discomfort (colposcopy) and morbidity (cervical biopsy/conization) as well as side effects such as hemorrhage, cervical stenosis, cervical incompetence, and preterm delivery [18], colposcopy is a valid tool for the detection of pre-invasive and early cervical carcinoma [19], and these concerns should be carefully balanced.

Persistent infection with high-risk HPV genotypes is necessary for the development of cervical cancer and its precursor lesion, cervical intraepithelial neoplasia grade 3 [20,21]. Therefore, cytology informed our study design. We formed a study group according to cytology findings and correlated these with colposcopy and histology findings. The most common referral cytology was HSIL. Papanicolaou smear demonstrated a sensitivity of 60–80% in detecting HSIL, although it was less sensitive in LSIL detection. Through the implementation of the Papanicolaou smear method and a well-organized cervical screening program, a reduction in invasive cervical cancer rates has been observed [22,23]. High-quality screening with cytology has also reduced mortality from squamous cell cervical cancer, which comprises 80–90% of all cervical cancers [24,25].

Our histology results are similar to those from different studies that have applied modern statistical techniques to evaluate the strength of the colposcopy impression and histology [26,27].

This study had some limitations, including the relatively small number of participants and the two different types of examinations (live and offline examination), which could have affected the accuracy of the images. Some studies have concluded that live examination induces more important variability [28]. Another source of bias was the definition of the three main colposcopic findings. Considering the great variation in the lesions and the lesional polymorphism on the same cervix, placing different aspects into one category may be problematic. The lack of data regarding the HPV and HPV vaccinations status of the patients represents another limitation of the study.

Our study underlines the importance of cervical screening and is in accordance with international programs to reduce CC incidence. For instance, according to the publication of the 2018 FIGO Cancer Report, giant strides have been made in the global effort to reduce the burden of cervical cancer, with the World Health Organization (WHO) rolling out a global strategy for cervical cancer elimination, aiming for implementation by 2030. Screening has seen major advances with the wider implementation of HPV testing [29]. Most importantly, in 2020, the ASCCP began to endorse any type cervical cancer screening for the secondary prevention of cervical cancer and recommends interventions that improve screening for those who are underscreened or unscreened [30].

## 5. Conclusions

Colposcopy remains an essential tool for the diagnosis of premalignant cervical lesions, particularly when limited resources make HPV detection unavailable. Our study indicates a good correlation between colposcopic and pathological findings. The standardization of the protocol used in this study resulted in insignificant interobserver variability, even during offline examination, and can serve as support for further postgraduate teaching.

## Figures and Tables

**Table 1:** Correspondence between colposcopic findings and cytology results obtained by evaluators 1 and 2.

Evaluator 1					
		Colposcopy Findings			Total
		HG	LG	Invasion	
Cytology	HSIL	45	29	4	78
	LSIL	2	34	0	36
	ASCH	5	7	0	12
	ASC-US-LSIL	0	2	0	2
Total		52	72	4	128
Evaluator 2					
		Colposcopy findings			Total
		invasion	LG	HG	
Cytology	HSIL	11	33	34	78
	LSIL	0	32	4	36
	ASCH	0	9	3	12
	ASC-US-LSIL	0	2	0	2
Total		11	76	41	128

HSIL: high-grade squamous intraepithelial lesion; LSIL: low-grade squamous epithelial lesion; ASC-H: atypical squamous cells for which a high-grade lesion cannot be excluded; ASC-US: atypical squamous cells of undetermined significance; LG: low-grade; HG: high-grade.

**Table 2:** Correspondence between the two colposcopic examiners.

Both Evaluators					
Count					
		Colposcopy 2			Total
		Invasion	LG	HG	
Colposcopy 1	HG	9	12	31	52
	LG	1	64	7	72
	invasion	1	0	3	4
Total		11	76	41	128
Pathology * Colposcopy 1 Crosstabulation					
Evaluator 1					
		Colposcopy 1			Total
		HG	LG	invasion	
Pathology	LG	16	66	0	82
	HG	25	5	2	32
	carcinoma	11	1	2	14
Total		52	72	4	128
Pathology * Colposcopy 2 Crosstabulation					
Evaluator 2					
		Colposcopy 2			Total
		invasion	LG	HG	
Pathology	LG	2	68	12	82
	HG	3	7	22	32
	carcinoma	6	1	7	14
Total		11	76	41	128

LG: low-grade; HG: high-grade.

**Table 3:** Correspondence between cytology and pathology.

Cytology * Pathology Crosstabulation					
Count					
		Pathology			Total
		LG	HG	Carcinoma	
Cytology	HSIL	39	26	13	78
	LSIL	33	3	0	36
	ASCH	8	3	1	12
	ASC-US-LSIL	2	0	0	2
Total		82	32	14	128

## Abbreviations

ASCCP: American Society for Colposcopy and Cervical Pathology; ASC-H: atypical squamous cells for which a high-grade lesion cannot be excluded; ASC-US: atypical squamous cells of undetermined significance; CI: confidence interval; HSIL: high-grade squamous intraepithelial lesion; HPV: human papillomavirus; LSIL: low-grade squamous epithelial lesion, cervical cytology.
